# gprofiler2 -- an R package for gene list functional enrichment analysis and namespace conversion toolset g:Profiler

**DOI:** 10.12688/f1000research.24956.2

**Published:** 2020-11-17

**Authors:** Liis Kolberg, Uku Raudvere, Ivan Kuzmin, Jaak Vilo, Hedi Peterson

**Affiliations:** 1 Institute of Computer Science, University of Tartu, Tartu, Tartumaa, 51009, Estonia

**Keywords:** g:Profiler, R package, functional enrichment analysis, identifier mapping, Gene Ontology, pathways

## Abstract

g:Profiler ( https://biit.cs.ut.ee/gprofiler) is a widely used gene list functional profiling and namespace conversion toolset that has been contributing to reproducible biological data analysis already since 2007. Here we introduce the accompanying R package, **gprofiler2**, developed to facilitate programmatic access to g:Profiler computations and databases via REST API. The **gprofiler2** package provides an easy-to-use functionality that enables researchers to incorporate functional enrichment analysis into automated analysis pipelines written in R. The package also implements interactive visualisation methods to help to interpret the enrichment results and to illustrate them for publications. In addition, **gprofiler2** gives access to the versatile gene/protein identifier conversion functionality in g:Profiler enabling to map between hundreds of different identifier types or orthologous species. The **gprofiler2** package is freely available at the CRAN repository.

## Introduction

Interpretation of gene lists is a key step in numerous biological data analysis workflows, such as differential gene expression analysis and co-expression clustering of RNA-seq or microarray data. Usually this involves associating these gene lists with previous knowledge from well curated data sources of biological processes and pathways. However, as the knowledge bases are constantly changing, keeping the associations up to date requires careful data management. Handling numerous databases, especially when using different gene identifier types, can be a very time-consuming process for researchers.

g:Profiler ( https://biit.cs.ut.ee/gprofiler) is a popular web toolset that helps to handle gene lists from various biological and biomedical studies of more than 600 species and strains, including vertebrates, plants, fungi, insects and parasites ^1, 2^. g:Profiler’s best known functionality is the over-representation analysis to identify significantly enriched biological functions and pathways obtained from well established data sources which include, among others, Gene Ontology (GO) ^3^, KEGG ^4^ and Reactome ^5^. The information about genes, identifier types and GO term associations in g:Profiler is mostly based on Ensembl databases ^6^ including data from Ensembl Genomes, fungi, plants and metazoa specific versions of Ensembl. g:Profiler follows Ensembl’s quarterly update cycle while keeping the access to previous data versions as archives for reproducibility. The parasite specific data is included from WormBase ^7^.

Providing users with fast and easy access has been the main goal of g:Profiler developers. Since 2007, g:Profiler has been in constant development and with the recent update in 2019 a new accompanying R package, **gprofiler2**, was developed ^8^. The R package relies on the g:Profiler REST API requests providing an easy programmatic access to the same functionalities as in the web tool without performing heavy computations and mappings in R. While there are other popular R packages for functional enrichment analysis, such as topGO ^9^ and clusterProfiler ^10^, **gprofiler2** provides access to numerous annotation data sources with a single query without requiring to download any of these sources to a local computer. Furthermore, the mapping between different gene identifiers is automatic and the input can be a mixed list of identifiers. g:Profiler’s continuous development and flexibility of usage has been recognised by the European Life Science Infrastructure ELIXIR, which has selected it as one of its Recommended Interoperability Resources.

g:Profiler development team encourages and supports external tools and packages to use either **gprofiler2** package or the public API to be part of their workflows. For example, RCAS Bioconductor package ^11^ includes **gprofiler2** for functional analysis of transcriptomic regions detected by different high-throughput experiments. Single-cell mapper package ( scMappR) ^12^ analyses cell-type specific gene lists with **gprofiler2**. OmnipathR ^13^ suggests using **gprofiler2** for enrichment analysis of protein complexes. Gene Co-expression Network analysis pipeline ( GWENA) uses **gprofiler2** in their pipeline for functional enrichment of co-expressed gene modules. A Nextflow differential gene expression analysis pipeline includes **gprofiler2** for pathway analysis.

Here we demonstrate how to conveniently incorporate the **gprofiler2** R package into bioinformatics analysis pipelines using differential gene expression analysis as an example.

## Methods

### Implementation

Inherently, **gprofiler2** ^8^ is a collection of wrapper functions in R that simplify sending POST requests to the g:Profiler REST API using the RCurl package ^14^. This means that all the annotation data sources and computations are centralised in a single well-maintained server and therefore the results from both the web tool and R package are guaranteed to be identical. Relying on the central API also simplifies the maintenance of the g:Profiler interfaces and enables the R users to get access to the most up-to-date data without having to download the heavy annotation data files to their own devices. At the same time, g:Profiler respects users’ privacy and does not store input gene lists unless the user explicitly requests their data to be stored for future reference via dedicated short links (see section “Sending analysis from R to g:Profiler web interface”). 

There are four main API wrapper functions in **gprofiler2**:

- gost for functional enrichment analysis
- gconvert for mapping gene identifiers between different namespaces
- gorth for mapping orthologous genes across species
- gsnpense for mapping SNP rs-IDs to chromosome positions, genes and variant effects.

In addition to fetching the results from the API, **gprofiler2** uses the packages ggplot2 ^15^ and plotly ^16^ to provide visualisations for enrichment results that are similar to the web tool ones. Using ggplot2 allows users to customise the visualisations by adding or removing graphical layers, and to adjust the quality of images for publication.

This article was written using R version 3.6.1 (2019-07-05) and **gprofiler2** version 0.1.9.

### Operation

The **gprofiler2** R package is available from CRAN and works on R versions 3.5 and above. The package also includes a detailed vignette.

The package can be installed from CRAN:


# install from CRAN
install.packages("gprofiler2")
# load the package
library(gprofiler2)


### Input description

The most popular functionality of g:Profiler is functional enrichment analysis provided by the g:GOSt tool that performs over-representation analysis using hypergeometric test. This functionality is available in **gprofiler2** under the function gost. The required inputs for this function are a vector of gene identifiers, query, and the name of the corresponding organism which is constructed by concatenating the first letter of the genus name and the specific epithet, e.g. *hsapiens* for human genes. The full list of supported species and strains, 641 in total, is available on the g:Profiler web page.

The query vector can include mixed types of gene/protein identifiers, SNP rs-IDs, chromosomal intervals or term IDs. Accepting a mixture of IDs is a unique feature that skips time-consuming manual steps of converting between different identifier types required by other functional enrichment tools. However, in case of analysing numeric identifiers (e.g. Entrez IDs) the user should specify the namespace using the numeric_ns parameter. The same description of input query and organism holds for the three other functions in **gprofiler2**.


gostres = gost(query = c("X:1000:1000000", "rs17396340", "GO:0005005",
		           "ENSG00000156103", "NLRP1", "3837"),
               organism = "hsapiens",
               numeric_ns = "ENTREZGENE_ACC")



Several additional parameters in the gost function help to perform the analysis according to specific needs, including custom statistical options such as background definition, statistical significance threshold, method for multiple testing correction and testing for under-representation. Also, additional information like GO evidence codes and genes belonging to the intersection between the input list and the functional term is available.

### Annotation databases

g:Profiler’s in-house database includes only reliable annotation data sources that are regularly updated such as Gene Ontology (GO) ^3^, KEGG ^4^, Reactome ^5^, WikiPathways ^17^, miRTarBase ^18^, TRANSFAC ^19^, Human Protein Atlas ^20^, protein complexes from CORUM ^21^ and Human Phenotype Ontology ^22^. By default, all data sources in g:Profiler database are used for the analysis in **gprofiler2**, but a specific selection can be defined with the sources parameter of the gost function. In order to enable more flexibility, users can also use their own annotation data. The custom source can be uploaded in a Gene Matrix Transposed (GMT) file format. This feature is further described in the next section.

## Use case

Differential gene expression analysis determines lists of genes that show changes in expression between different conditions, cell types, time points, etc. Functional enrichment analysis using the **gprofiler2** package ^8^ helps to interpret these gene lists.

Here we demonstrate the main functionality of **gprofiler2** by following an analysis example from the existing RNA-seq Bioconductor workflow ^23^ that uses the popular DESeq2 package ^24^ for differential analysis. The example RNA-seq data are obtained from the airway package ^25^ that comprises experimental data where airway smooth muscle cells were treated with dexamethasone.


# installing Bioconductor packages
if (!requireNamespace("BiocManager", quietly = TRUE)) install.packages("BiocManager")
BiocManager::install(c("DESeq2","airway"))



library(DESeq2)
library(airway)
library(gprofiler2)



### Functional enrichment of differentially expressed genes

First, we will detect the list of genes that are differentially regulated when stimulated with dexamethasone and then we will use the function gost from **gprofiler2** to find the biological functions and pathways that are significantly enriched in this gene set.


# load the airway data
data(airway)
# construct the DESeqDataSet object
ddsMat = DESeqDataSetFromMatrix(countData = assay(airway),
			            colData = colData(airway),
			            design = ^~^ cell + dex)
# run DESeq2 pipeline
dds = DESeq(ddsMat)
# get the results
results = results(dds, contrast = c("dex", "trt", "untrt"),
                   alpha = 0.05, lfcThreshold = 1)
# keep only the significant genes
results_sig = subset(results, padj < 0.05)
# get the significant up-regulated genes
up = subset(results_sig, log2FoldChange > 0)
# get the significant down-regulated genes
down = subset(results_sig, log2FoldChange < 0)



# enrichment analysis
gp_up = gost(row.names(up), organism = "hsapiens")
gp_down = gost(row.names(down), organism = "hsapiens") 


The output of the gost function is a named list where the element result includes a data frame with the enriched functions and related statistics; and the element meta includes relevant metadata for reproducing these results.


head(gp_up$result)



##      query significant     p_value term_size query_size intersection_size
## 1  query_1        TRUE 0.001414822      9455        124                92
## 2  query_1        TRUE 0.003848846        14        124                 4
## 3  query_1        TRUE 0.003848846        14        124                 4
## 4  query_1        TRUE 0.006923191        16        124                 4
## 5  query_1        TRUE 0.006923191        16        124                 4
## 6  query_1        TRUE 0.012886939      1024        124                21
##     precision      recall    term_id source                            term_name
## 1  0.74193548 0.009730301 GO:0050896  GO:BP                 response to stimulus
## 2  0.03225806 0.285714286 GO:0010273  GO:BP         detoxification of copper ion
## 3  0.03225806 0.285714286 GO:1990169  GO:BP        stress response to copper ion
## 4  0.03225806 0.250000000 GO:0097501  GO:BP         stress response to metal ion
## 5  0.03225806 0.250000000 GO:0061687  GO:BP detoxification of inorganic compound
## 6  0.16935484 0.020507812 GO:0009725  GO:BP                   response to hormone
##    effective_domain_size source_order                parents
## 1                  17906        15742             GO:0008150
## 2                  17906         4576 GO:0061687, GO:1990169
## 3                  17906        29137 GO:0046688, GO:0097501
## 4                  17906        22329 GO:0006950, GO:0010038
## 5                  17906        18680             GO:0098754
## 6                  17906         4118 GO:0009719, GO:0010033


### Accounting for the order of genes in enrichment analysis

For cases where the list of interesting genes can be ranked by some biologically meaningful measure, such as P-value or fold change in differential analysis, g:Profiler provides an ordered query option that takes the ranking into account when performing enrichment tests. The testing is then performed iteratively, starting from the first gene and sequentially adding genes one by one. For every term, the smallest enrichment P-value is reported along with the corresponding gene list size. Consequently, for different terms the query size can vary, especially as the broader terms can be enriched for larger lists only. This option is very similar to the idea of the GSEA analysis method ^26^.

For example, to perform ordered query using **gprofiler2** we first rearrange the list of up-regulated genes based on the log _2_ fold change values so that the first gene in the list has the highest value. Next we use this ordered list as a query in the gost function and set the parameter ordered_query = TRUE.


# order genes by log2FC
up_ordered = up[order(up$log2FoldChange, decreasing = TRUE),]
# ordered enrichment analysis
gp_up_ordered = gost(row.names(up_ordered), organism = "hsapiens",
		        ordered_query = TRUE)
head(gp_up_ordered$result, 8)



##     query significant      p_value term_size query_size intersection_size
## 1 query_1        TRUE 0.0006617979        14         80         	   4
## 2 query_1        TRUE 0.0006617979        14         80        	   4
## 3 query_1        TRUE 0.0011951178        16         80         	   4
## 4 query_1        TRUE 0.0011951178        16         80          	   4
## 5 query_1        TRUE 0.0047065333        22         80          	   4
## 6 query_1        TRUE 0.0080566224        25         80          	   4
## 7 query_1        TRUE 0.0132789677       106        120        	   7
## 8 query_1        TRUE 0.0159695023       109        120            	   7
##    precision     recall    term_id source                            term_name
## 1 0.05000000 0.28571429 GO:1990169  GO:BP 	    stress response to copper ion
## 2 0.05000000 0.28571429 GO:0010273  GO:BP 	     detoxification of copper ion
## 3 0.05000000 0.25000000 GO:0097501  GO:BP	     stress response to metal ion
## 4 0.05000000 0.25000000 GO:0061687  GO:BP detoxification of inorganic compound
## 5 0.05000000 0.18181818 GO:0071294  GO:BP 	    cellular response to zinc ion
## 6 0.05000000 0.16000000 GO:0071280  GO:BP   	  cellular response to copper ion
## 7 0.05833333 0.06603774 GO:0003300  GO:BP 	       cardiac muscle hypertrophy
## 8 0.05833333 0.06422018 GO:0014897  GO:BP	      striated muscle hypertrophy
##   effective_domain_size source_order                parents
## 1 	   	     17906        29137 GO:0046688, GO:0097501
## 2 	             17906	   4576 GO:0061687, GO:1990169
## 3	             17906	  22329 GO:0006950, GO:0010038
## 4	             17906	  18680 	    GO:0098754
## 5	             17906 	  19684 GO:0010043, GO:0071248
## 6	             17906 	  19670 GO:0046688, GO:0071248
## 7	             17906	   1905 	    GO:0014897
## 8 	             17906	   5377 	    GO:0014896


The resulting data frame is in the same format as shown previously. Only the size of the query in the table can vary as the algorithm detects the most significant cutting point from the input gene list considering every function separately.

### Visualisation of functional enrichment results

Different visualisations are useful to summarise and interpret functional enrichment results. With the recent update, g:Profiler introduced an alternative way for visualising functional terms, a Manhattan plot. On this plot, the x-axis shows the terms and y-axis shows the enrichment P-values on − log _10_ scale. Each circle on this plot corresponds to a single term. The circles are colored according to the annotation source and size-scaled according to the total number of genes annotated to the corresponding term. The locations on the x-axis are always fixed and ordered in a way that the terms from the same GO subtree are located closer to each other. This helps to highlight different enriched GO sub-branches as they form peaks in the Manhattan plot and makes plots from different queries easily comparable. For the same reason, by default the values on the y-axis are capped to a maximum value of 16 that corresponds to P-value less than 10 ^−16^ . The same default threshold is also used in the statistical tests in R. This selection can be switched off to show the P-values in a wider scale range.

Interactive graphs are common in web tools and therefore the Manhattan plot in g:Profiler web interface also provides several interactive features to facilitate data exploration and enables to export the visualisations as high-quality image files. Mimicking the g:Profiler web interface, the Manhattan plot in **gprofiler2** is implemented in the function gostplot that uses the resulting object from the gost function as an input. As a unique feature, compared to other similar packages, the parameter interactive enables to switch between interactive plotly graph for browsing or static ggplot graph for saving as an image file. The parameter capped enables to turn off the upper limit of y-axis.


gostplot(gp_up, interactive = TRUE)


**Figure 1.**
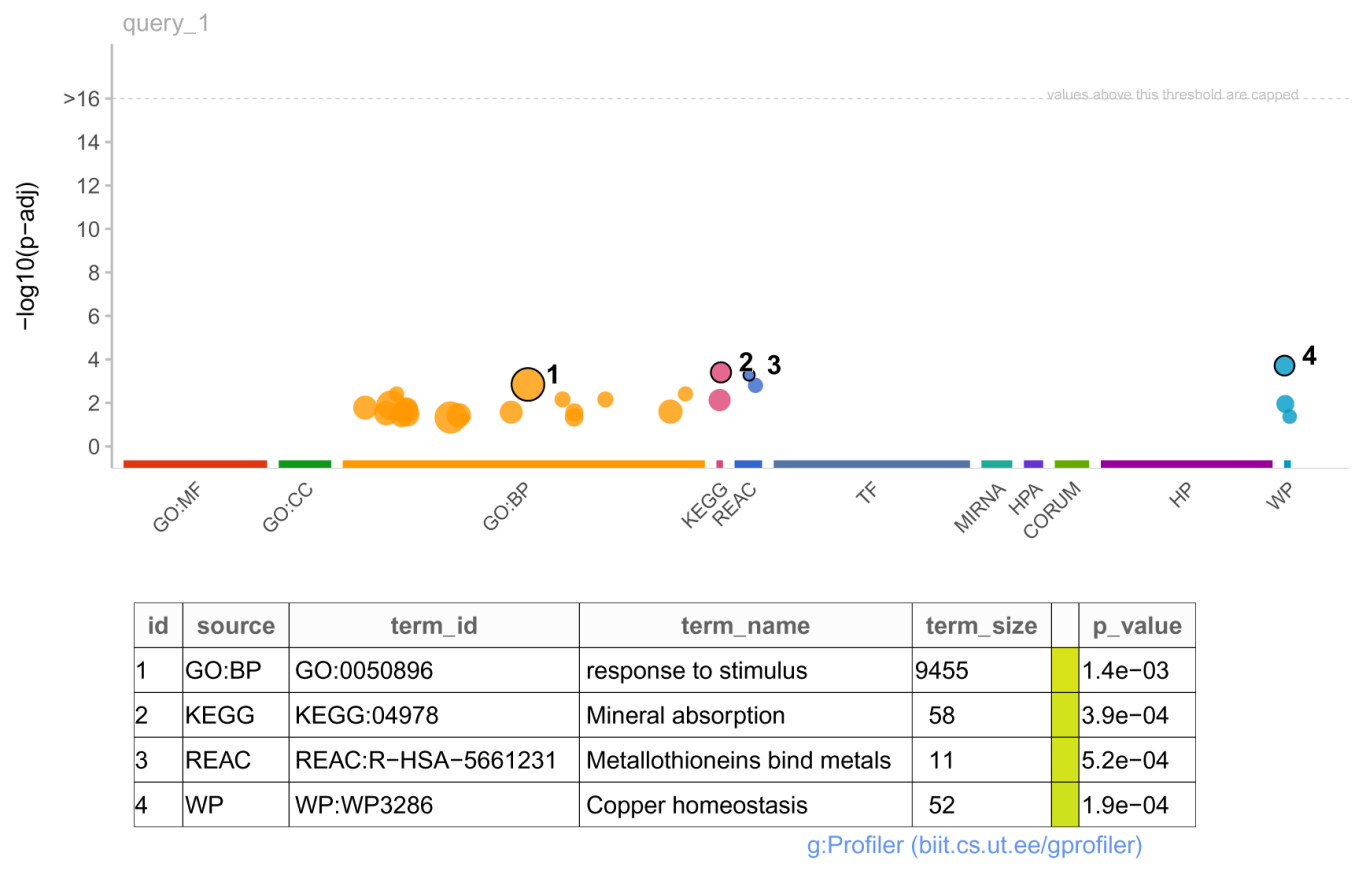
Manhattan plot of g:Profiler enrichment results.

After exploring the interactive graph and deciding on the story to tell about the results, the user can compose a publishable figure that highlights the most important terms using the function publish_gostplot and defining the relevant terms in the parameter highlight_terms. The chosen terms are indicated with numbers on the plot and corresponding statistics are shown in the table below the Manhattan plot. For example, the enrichment results for up-regulated genes are shown in Figure 1. The Manhattan plot can be saved as an image file (PNG, PDF, JPEG, etc) specified by the filename parameter.


p1 = gostplot(gp_up, interactive = FALSE)
publish_gostplot(p1, highlight_terms = c("GO:0050896", "KEGG:04978",
					     "REAC:R-HSA-5661231", "WP:WP3286"))


As the resulting plot is a standard ggplot object, it is easy to further customise the graphs by adding graphical layers or textual annotations.

### Analysing multiple gene lists

Above we were analysing the up- and down-regulated gene lists separately, but the gost function also works with a (named) list of multiple gene vectors that enables to keep all the results in a single object and to easily compare different groups.


multi_gp = gost(list("up-regulated" = row.names(up),
	  "down-regulated" = row.names(down)))


In this case, the resultant data frame is in a so-called “long format” where the column query includes the names of corresponding input vectors to differentiate between them. The alternative is to set multi_query = TRUE which, in case of multiple gene lists, returns results as a comparison table in a “wide format”. That is, the rows are concatenated by terms and query statistics are shown in cells as vectors, e.g. the p_values column includes a vector of corresponding P-values from all the input queries, even the insignificant ones.

**Figure 2.**
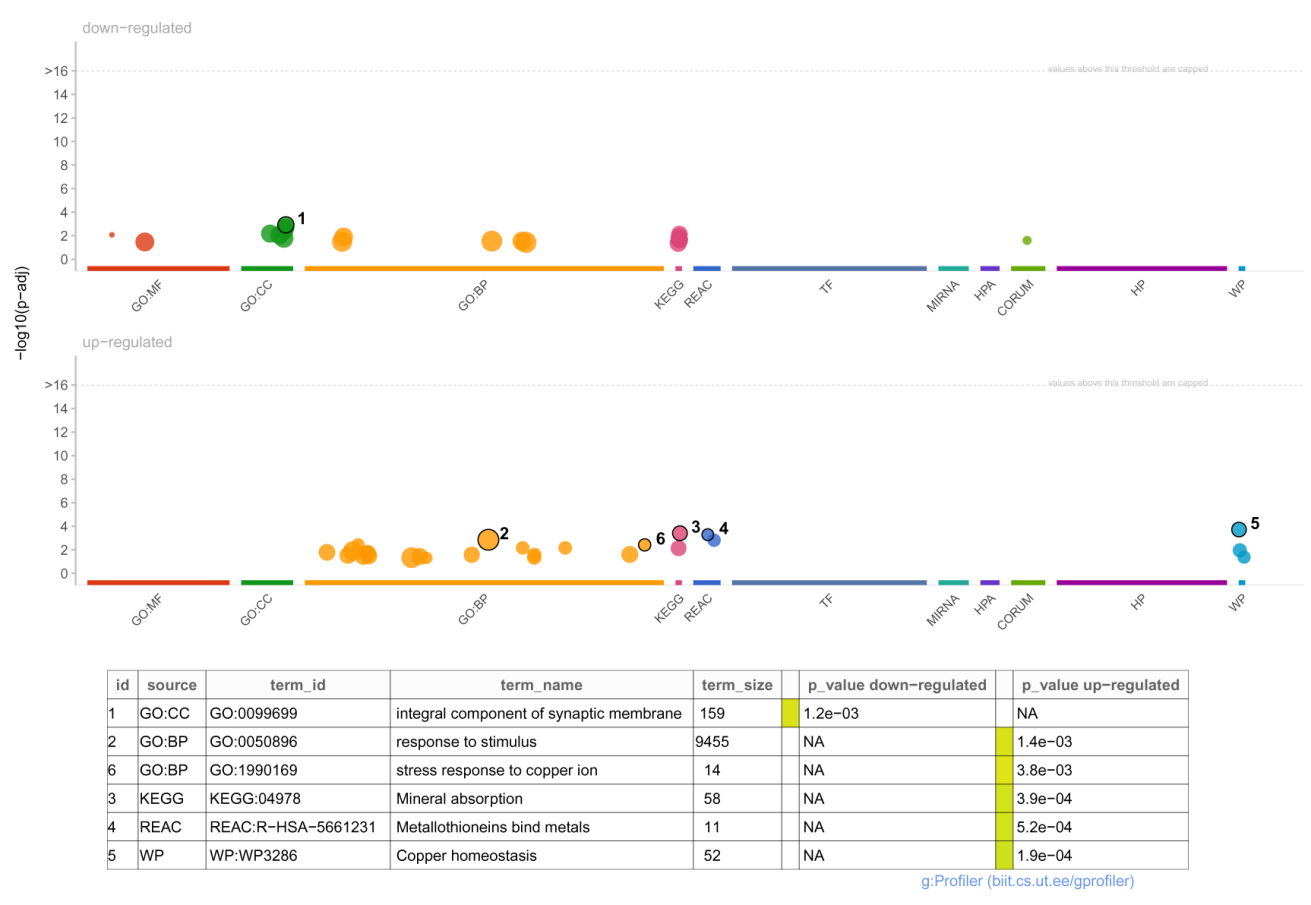
Visualisation of g:Profiler enrichment results to compare multiple gene lists.

Results from multiple gene lists can also be used for plotting. The function gostplot detects the case of multiple queries and plots the Manhattan plots under each other for comparison. The example enrichment results are shown in Figure 2.


p2 = gostplot(multi_gp, interactive = FALSE)
publish_gostplot(p2, highlight_terms = c("GO:0099699", "GO:0050896", "KEGG:04978",
					       "REAC:R-HSA-5661231", "WP:WP3286",
					       "GO:1990169"))


### Sending analysis from R to g:Profiler web interface

The same enrichment results can also be viewed in the g:Profiler web tool. The user can generate a dedicated short-link by setting the parameter as_short_link = TRUE in the gost function which then returns the short-link to g:Profiler web tool instead of a data frame. This is a useful feature for sharing the results easily with colleagues or to accompany a publication without the peers having to run the full analysis code in R.


multi_gp_link = gost(list("up-regulated" = row.names(up),
	   "down-regulated" = row.names(down)), as_short_link = TRUE)


In this case, the variable multi_gp_link is a character string that corresponds to a stable short-link to these enrichment results: https://biit.cs.ut.ee/gplink/l/0wgtcERnQT. We also note that the input gene lists will be stored in a database to provide short-link access. 

### Mapping between gene identifiers with gconvert

Another common but tedious task in handling gene lists is mapping between different identifiers. The function gconvert helps to easily solve this issue and translates the given input identifiers to some other user defined namespace together with gene names and descriptions. The function is able to map between at least 30 different namespaces for more than 190 species. All available namespaces for different organisms are listed on the g:Profiler page.

As an example we will convert the Ensembl IDs in our differential expression results to numeric Entrez IDs with gconvert. The function takes a vector of gene identifiers as an input and returns a data frame that includes a column with target identifiers together with the names and descriptions for the input genes.


results_genes = gconvert(row.names(results), organism = "hsapiens",
			     target = "ENTREZGENE_ACC", filter_na = FALSE)
head(results_genes)



##   input_number           input target_number  target     name
## 1 		1 ENSG00000000003           1.1    7105   TSPAN6
## 2 		2 ENSG00000000005           2.1   64102     TNMD
## 3 		3 ENSG00000000419           3.1    8813     DPM1
## 4 		4 ENSG00000000457           4.1   57147    SCYL3
## 5 		5 ENSG00000000460           5.1   55732 C1orf112
## 6 		6 ENSG00000000938           6.1    2268      FGR
## 										        description
## 1 						  tetraspanin 6 [Source:HGNC Symbol;Acc:HGNC:11858]
## 2 						    tenomodulin [Source:HGNC Symbol;Acc:HGNC:17757]
## 3 dolichyl-phosphate mannosyltransferase subunit 1, catalytic [Source:HGNC Symbol;Acc:HGNC:3005]
## 4 				       SCY1 like pseudokinase 3 [Source:HGNC Symbol;Acc:HGNC:19285]
## 5 			    chromosome 1 open reading frame 112 [Source:HGNC Symbol;Acc:HGNC:25565]
## 6 		  FGR proto-oncogene, Src family tyrosine kinase [Source:HGNC Symbol;Acc:HGNC:3697]
## 	     namespace
## 1 ARRAYEXPRESS,ENSG
## 2 ARRAYEXPRESS,ENSG
## 3 ARRAYEXPRESS,ENSG
## 4 ARRAYEXPRESS,ENSG
## 5 ARRAYEXPRESS,ENSG
## 6 ARRAYEXPRESS,ENSG


The users can add this information to the differential expression results data frame and save it to a tab separated text file to include as a supplementary file in their article, for example.


results_df = as.data.frame(results)
results_df$Ensembl_id = row.names(results_df)
results_df = results_df[order(results_df$padj),]

# add the gene names
results_df = merge(results_df,
		   results_genes[,c("input", "target", "name", "description")],
		      by.x = "Ensembl_id", by.y = "input")

# save the results to a tsv file
write.table(results_df, file = "DESeq2_results.tsv", sep = "\t",
              quote = F, row.names = F)


### Using custom annotations

While g:Profiler enables to analyse genes from numerous organisms using high-quality annotation databases, there is still a need for custom data functionality for researchers interested in non-model organisms, that are not annotated in the Ensembl database, or in some specific, not so widespread annotation resource. In g:Profiler, this is solved by enabling users to upload custom annotation files in the GMT file format, which is essentially a tab delimited text file where every row describes a function by its identifier, description, and the genes annotated in this function. Here it is important to note that in case of custom annotation files, all the identifiers not present in the GMT file will be ignored in the analysis.

For example, to use the gene-disease association data from the DisGeNET database ^27^ for enrichment analysis, the user can upload the GMT file in R using the upload_GMT_file function that returns a unique token for the file which can then be used as a value for the organism argument in the gost function.

First, we use R utility function download.file to download an annotation GMT file from DisGeNET into a file in the working directory and name it “DisGeNET.gmt”.


# download the GMT file from DisGeNET
gmturl = file.path("http://www.disgenet.org",
		     "static/disgenet_ap1/files/downloads/gmt_files",
		     "disgenet.curated.v7.symbols.gmt")
download.file(url = gmturl, destfile = "DisGeNET.gmt")


Now, when we have the file in our local environment, we can upload it to g:Profiler with the upload_GMT_file function.


token = upload_GMT_file(gmtfile = "DisGeNET.gmt")
# save this token to your notes for enrichment analysis


The result of this upload is a unique token (in this case "gp_goJy_Ej2J_rPc") which should be saved by the user for future use. In order to find the enriched diseases in our gene list, we will use the token as a value for the organism in the gost function. As the DisGeNET database file includes gene symbols and not Ensembl identifiers, we first use gconvert to map our Ensembl IDs to gene names and use these as the input for the enrichment analysis.


up_names = gconvert(row.names(up))
down_names = gconvert(row.names(down))

custom_gp = gost(list("up-regulated" = up_names$name,
                       "down-regulated" = down_names$name),
      organism = "gp__goJy_Ej2J_rPc")


**Figure 3.**
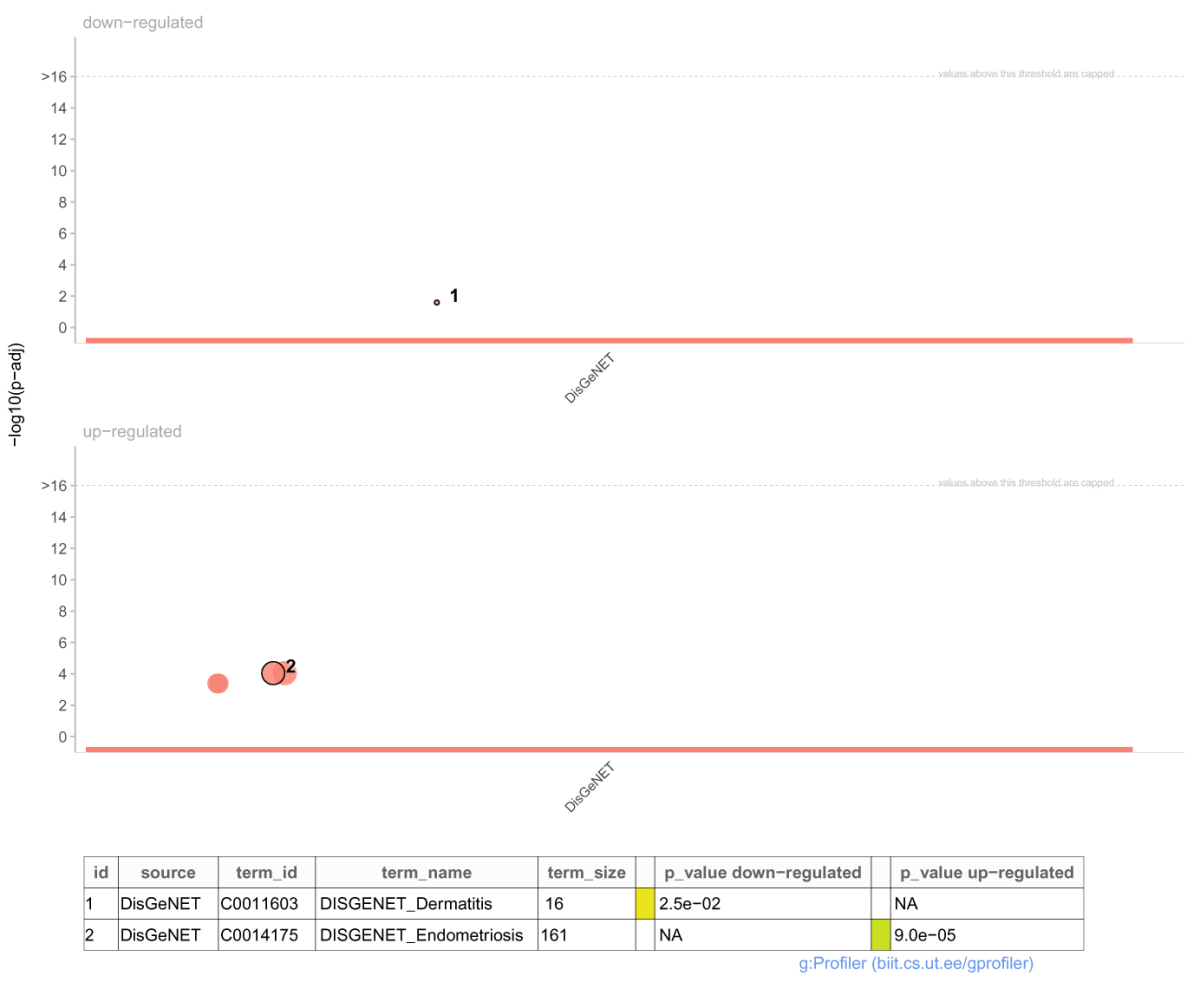
Manhattan plot of g:Profiler enrichment results using DisGeNET database loaded from a custom GMT file.

The custom data source results can also be plotted using the Manhattan plots ( Figure 3). In this case, the term position on the x-axis is defined by the order in the GMT file.


p = gostplot(custom_gp, interactive = FALSE, pal = list("DisGeNET" = "salmon"))
pp = publish_gostplot(p, highlight_terms = c("C0011603", "C0014175"))


As the **gprofiler2** R package and the web tool are in sync, this token will also work for the analysis in the web tool and can be inserted under the section “Bring your own data (Custom GMT)”. And vice versa, the token obtained from the web tool will work in the R package without uploading the data again. Thus, in order to analyse multiple gene lists with the same data source, the user needs to upload the file only once and can use the given token from then on. Furthermore, analysing multiple custom sources at once is enabled with the upload of a ZIP archive that includes multiple GMT files. GMT file names are used as the names for the data sources in the results and colored independently in the Manhattan plot.

### Mapping orthologous genes with gorth

Sometimes, in order to further investigate the interesting set of differential genes in human, researchers need to perform additional experiments on model organisms such as mice. This requires finding the corresponding orthologs of these interesting genes from other species. Another use for orthologous genes is the possibility to transfer the extensive knowledge from well studied organisms to less studied species.

Mapping orthologous genes between species in g:Profiler is enabled by the g:Orth tool and in the **gprofiler2** package the access is wrapped into the function gorth. The function works very similarly to gconvert, only in this case the user has to define corresponding source_organism and target_organism. For example, the following code maps the detected up-regulated gene identifiers to corresponding mice genes.


mouse_genes = gorth(row.names(up), source_organism = "hsapiens",
                        target_organism = "mmusculus")
head(mouse_genes)



##   input_number           input      input_ensg ensg_number ortholog_name
## 1            1 ENSG00000035664 ENSG00000035664       1.1.1         Dapk2
## 2            2 ENSG00000046653 ENSG00000046653       2.1.1         Gpm6b
## 3            3 ENSG00000060718 ENSG00000060718       3.1.1       Col11a1
## 4            4 ENSG00000068383 ENSG00000068383       4.1.1        Inpp5a
## 5            5 ENSG00000068831 ENSG00000068831       5.1.1       Rasgrp2
## 6            6 ENSG00000070404 ENSG00000070404       6.1.1         Fstl3
##        ortholog_ensg
## 1 ENSMUSG00000032380
## 2 ENSMUSG00000031342
## 3 ENSMUSG00000027966
## 4 ENSMUSG00000025477
## 5 ENSMUSG00000032946
## 6 ENSMUSG00000020325
##                                                                  description
## 1      death-associated protein kinase 2 [Source:MGI Symbol;Acc:MGI:1341297]
## 2                        glycoprotein m6b [Source:MGI Symbol;Acc:MGI:107672]
## 3               collagen, type XI, alpha 1 [Source:MGI Symbol;Acc:MGI:88446]
## 4 inositol polyphosphate-5-phosphatase A [Source:MGI Symbol;Acc:MGI:2686961]
## 5        RAS, guanyl releasing protein 2 [Source:MGI Symbol;Acc:MGI:1333849]
## 6                     follistatin-like 3 [Source:MGI Symbol;Acc:MGI:1890391]


This function returns a data frame that includes the input and target identifiers, and also the ortholog names and descriptions.

### Integrating with external tools for visualisations

Since the output of the gost function is stored in a standard data frame format, it is easy to alter it for custom visualisations using ggplot2, enrichplot ^28^, clusterProfiler ^10^ or any other similar package. Here we demonstrate how to convert the results from multiple gene lists into enrichResult and compareClusterResult objects required by the visualisations methods implemented in the enrichplot package. Similar approach also works for a single query.


# installing additional packages
# if (!requireNamespace("BiocManager", quietly = TRUE))
# install.packages("BiocManager")
BiocManager::install(c("clusterProfiler", "enrichplot", "DOSE"))



# loading the additional packages
library(clusterProfiler)
library(enrichplot)
library(DOSE) # needed to convert to enrichResult object


up_names = gconvert(row.names(up))
down_names = gconvert(row.names(down))

# enrichment analysis using gene names
multi_gp = gost(list("up-regulated" = up_names$name,
          "down-regulated" = down_names$name), multi_query = FALSE, evcodes = TRUE)

# modify the g:Profiler data frame
gp_mod = multi_gp$result[,c("query", "source", "term_id",
                                "term_name", p_value", query_size", 
                                "intersection_size", "term_size", 
                                "effective_domain_size", "intersection")]
gp_mod$GeneRatio = paste0(gp_mod$intersection_size,  "/", gp_mod$query_size)



gp_mod$BgRatio = paste0(gp_mod$term_size, "/", gp_mod$effective_domain_size)
names(gp_mod) = c("Cluster", "Category", "ID", "Description", "p.adjust", 
                    "query_size", "Count", "term_size", "effective_domain_size", 
                    "geneID", "GeneRatio", "BgRatio")
gp_mod$geneID = gsub(",", "/", gp_mod$geneID)
row.names(gp_mod) = gp_mod$ID

# define as compareClusterResult object
gp_mod_cluster = new("compareClusterResult", compareClusterResult = gp_mod)

# define as enrichResult object
gp_mod_enrich  = new("enrichResult", result = gp_mod)


**Figure 4.**
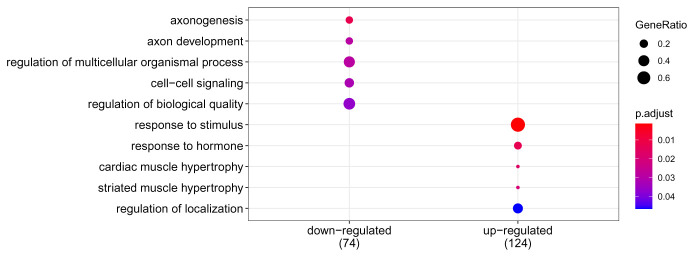
Dot plot of g:Profiler enrichment results using *enrichplot*.

After creating an instance of the enrichResult or compareClusterResult (for multiple gene lists) class from the gost result, this object can be used as an input for the visualisation functions from enrichplot and clusterProfiler that are suitable for over-representation analysis such as dotplot, barplot, cnetplot, upsetplot, emapplot, etc. Figure 4 shows the dot plot for results in a compareClusterResult object.



enrichplot::dotplot(gp_mod_cluster)


**Figure 5.**
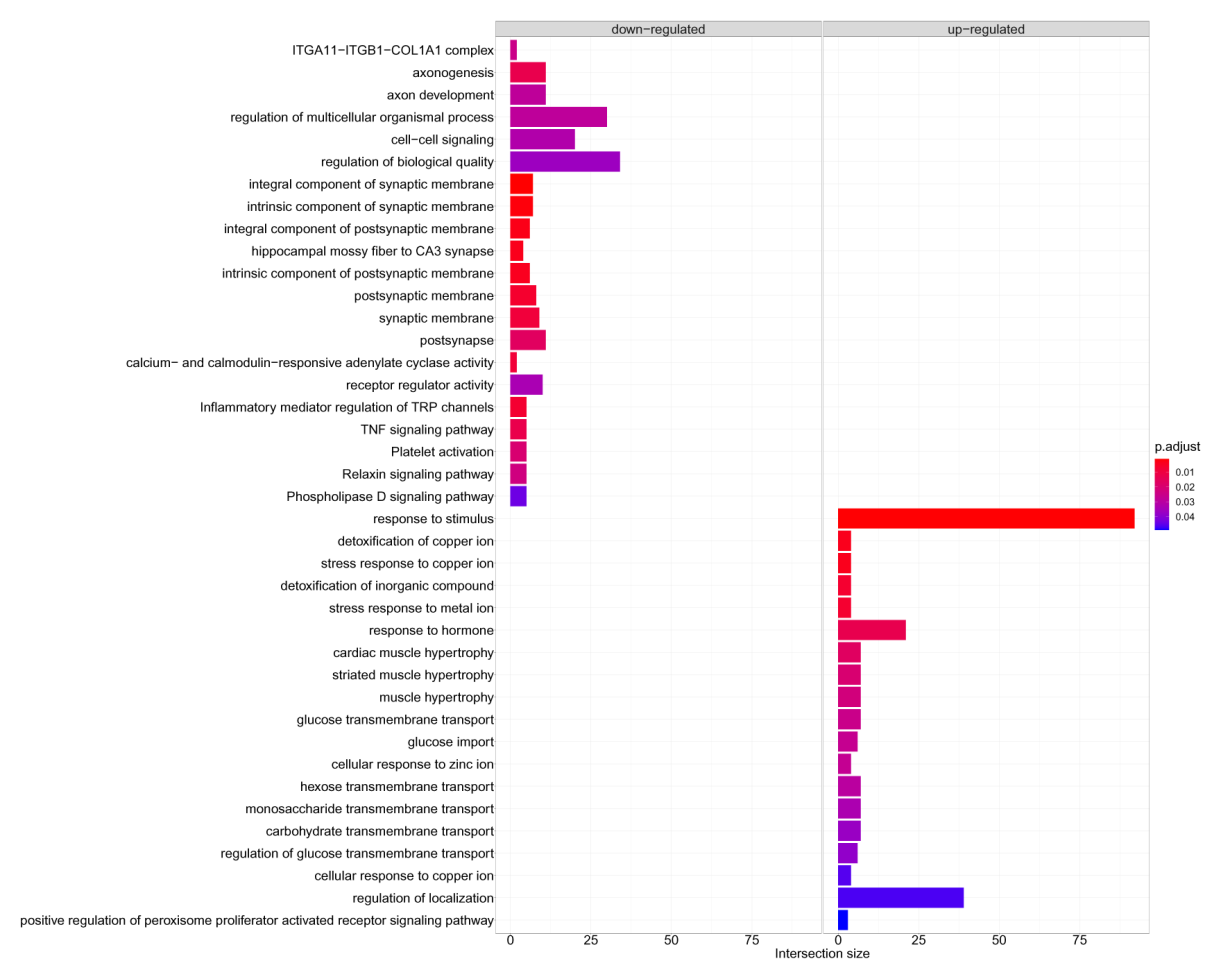
Bar plots of g:Profiler enrichment results using *enrichplot*.

As these plots are ggplot objects, using ggplot2 layers allows further customisation of the visualisations as shown in Figure 5.


barplot(gp_mod_enrich, showCategory = 40, font.size = 16) + 
  ggplot2::facet_grid(^~^Cluster) +
  ggplot2::ylab("Intersection size")


In order to use the browseKEGG function to open KEGG pathway browser, the pathway IDs should be transformed according to the organism. In case of human pathways, the prefix *KEGG* should be replaced with *hsa*. The full list of organisms and their prefixes is available from the KEGG home page.


gp_mod$ID[gp_mod$Category=="KEGG"] = gsub("KEGG:", "hsa",
	                                       gp_mod$ID[gp_mod$Category=="KEGG"], "")
row.names(gp_mod) = gp_mod$ID
# define as enrichResult object
gp_mod_enrich = new("enrichResult", result = gp_mod)



clusterProfiler::browseKEGG(gp_mod_enrich, pathID = "hsa04750")


This command will open the KEGG browser page for the pathway Inflammatory mediator regulation of TRP channels.

### Using g:Profiler results in EnricmentMap

The functional enrichment results from the gost function can be modified in order to save them into a Generic Enrichment Map (GEM) file format that is compatible with the EnrichmentMap application in Cytoscape ^29^. This app helps to visualise enrichment results as a highly customisable network where nodes represent enriched terms and edges represent their mutual overlap.

In case of a single query, the GEM file can be generated with the following lines of code. The parameter value evcodes = TRUE is important for obtaining the intersection column with corresponding gene IDs in the query that are annotated to the term.


gostres = gost(query = list("up-regulated" = row.names(up)),
                  evcodes = TRUE, multi_query = FALSE,
                  sources = c("GO", "REAC", "MIRNA", "CORUM", "HP", "HPA", "WP"))



gem = gostres$result[,c("term_id", "term_name", "p_value", "intersection")]



colnames(gem) = c("GO.ID", "Description", "p.Val", "Genes")
gem$FDR = gem$p.Val
gem$Phenotype = "+1"
gem = gem[,c("GO.ID", "Description", "p.Val", "FDR", "Phenotype", "Genes")]
# saving the GEM file
write.table(gem, file = "gProfiler_gem.txt", sep = "\t", quote = F, row.names = F)


In the EnrichmentMap the user can set the “Analysis Type” parameter as *Generic/gProfiler* and upload the required files: GEM file with enrichment results (input field “Enrichments”) and GMT file that defines the annotations (input field “GMT”). Both of these files have to include gene identifiers from the same namespace for the EnrichmentMap to work.

The GMT files used by g:Profiler are downloadable from the web page under the “Data sources” section. Only the GMT files of KEGG and Transfac are not available as the sharing is restricted by data source licenses.


# download GMT file for these results
download.file(
  url = "http://biit.cs.ut.ee/gprofiler/static/gprofiler_full_hsapiens.ENSG.gmt")


### Reproducibility

The demand for better reproducibility of computational analyses is constantly growing ^30^. In bioinformatics analysis, many different tools and databases are combined in order to detect relevant findings. This adds an extra layer of complexity which often leads to reproducibility issues. Because of this, since 2011 all the past releases of g:Profiler are maintained and kept usable to ensure reproducibility and transparency of enrichment analysis results. The users can cite the exact extract of the annotation database and the state of the implementation by stating the version number in their research. In **gprofiler2**, this is available, along with other query information, from the metadata of gost enrichment results:


# get g:Profiler version
multi_gp$meta$version



## [1] "e99_eg46_p14_f929183"


The g:Profiler specific version number notes that the results were obtained using the state of the database that includes data from Ensembl release 99, Ensembl Genomes release 46 and WormBase ParaSite release 14, among other sources, and the g:Profiler codebase with the Git revision number f929183. The version number together with the details of applied parameters (available from multi_gp$meta$query_metadata) is enough to reproduce the enrichment results in g:Profiler. A more detailed information about the data source versions in a given g:Profiler version is available from the g:Profiler web page https://biit.cs.ut.ee/gprofiler under the link “Show data versions” in the “Data sources” section.

In order to reproduce the results obtained with a specific version, one can change the data version using the function set_base_url:


set_base_url("http://biit.cs.ut.ee/gprofiler_archive3/e99_eg46_p14")


All the past versions and their URLs are available at https://biit.cs.ut.ee/gprofiler/page/archives. **gprofiler2** works with versions e94_eg41_p11 and higher, earlier versions are still accessible using the deprecated R package **gProfileR**.

Function set_base_url also gives access to the most recent developments and data updates of g:Profiler available at the Beta version:


set_base_url("http://biit.cs.ut.ee/gprofiler_beta")


In order to determine the current g:Profiler URL used for the analysis one can use the function get_base_url:


get_base_url()



## [1] "http://biit.cs.ut.ee/gprofiler_beta"


## Conclusion

We presented the **gprofiler2** R package ^8^ that is one of the programmatic access points to the widely used g:Profiler web toolset for gene list functional enrichment analysis and identifier conversion. This package enables effective integration of g:Profiler functionalities in various bioinformatics pipelines and tools written in R without the need of searching and downloading several data files. The suite of functions in **gprofiler2** are implemented with the importance of analysis reproducibility and interoperability with other tools in mind. In addition, the package provides a way to easily create or customise the enrichment plots using the existing visualisation packages in R. For the researchers who prefer to perform their computational analysis pipelines through the web, we have wrapped the **gprofiler2** package as a tool for the Galaxy platform ^31^.

It is important to note that using **gprofiler2** for functional enrichment analysis is not limited to the use case of differential gene expression analysis. The package is useful whenever there is a set of genes/proteins/SNPs the user wants to characterise with biological functions or to convert to another namespace.

## Data availability

All data underlying the results are available as part of the article and no additional source data are required.

## Software availability

**R package gprofiler2 is available from CRAN:** https://cran.r-project.org/package=gprofiler2.

**Source code available from:** https://gl.cs.ut.ee/biit/r-gprofiler2.

**Archived source code at time of publication:** https://doi.org/10.5281/zenodo.3919795 ^8^.

**License:** GNU General Public License v2.0.

## Author information

LK, UR and IK implemented the package. LK and HP wrote the article. HP and JV supervised the development.
