# Supplementary material for: gprofiler2 -- an R package for gene list functional enrichment analysis and namespace conversion toolset g:Profiler
Source: F1000Res. 2020 Nov 17;9:ELIXIR-709. Originally published 2020 Jul 15. [Version 2] doi: 10.12688/f1000research.24956.2 (PMC7859841; doi:10.12688/f1000research.24956.2)
Supplement: Peer review history

# Peer review: Reviewer response for version 2

**DOI:** 10.5256/f1000research.29655.r76696

**Published:** 2021-02-03

**Authors:** Ewy Mathe

**Affiliations:** 1 National Center for Advancing Translational Sciences/National Institutes of Health, Bethesda, MD, USA

Authors present an R package, gprofiler2, which provides programmatic access to the g:Profiler software, including underlying databases. Functionalities of the package include the ability to map genes to pathways (allowing different types of IDs as input), pathway enrichment analyses and visualizations, adding customized pathway annotations, and mapping orthologous genes between species. The implementation of gprofiler 2 is in sync with the g:Profiler web application and care has been taken to ensure reproducibility of results between both software (e.g. working off the same version of the underlying REST API). 

 Some comments for v2 are:

- While gprofiler2 also supports the mapping of SNP rs-IDs, this functionality was not further described in this version. Should it be added?
- The use of the namespace feature in the gost() function is unclear. What are the possible entries that are accepted for the “numeric_ns” parameter? The default seems to be “ENTREZGENE_ACC”?
- In gconvert(), it would be useful to provide a list of possible target types that are accepted by the target parameter, in both the paper and function man page.
- In the section on “Using custom annotations”, authors could consider clarifying that the custom annotations provide alternate pathways or gene lists and potentially changing the section name to “Using custom pathway/gene list annotations".

Is it possible to add a custom pathway annotation to existing annotations?

  

- For the pathway enrichment analyses performed, is there an option for multiple comparison adjustments? 
- In the code block under the “Integrating with external tools for visualisations” section, there are issues with the quotations:

gp_mod = multi_gp$result[,c("query", "source", "term_id",

 "term_name", **"**p_value", **"**query_size",

 "intersection_size **"**, "term_size",

 "effective_domain_size", "intersection")]

 Also, in this section title “visualisations”, there is a typo, should be spelled “visualizations”

  

- In the section “Using g:Profiler results in EnricmentMap”, it would be useful to show example code on how to draw a network of the enrichment results using RCytoskcape or at least some links to tutorials in using EnrhcihmentMap. 

Also, there is a typo in “Using g:Profiler results in EnricmentMap”, which should be spelled EnrichmentMap

Are the conclusions about the tool and its performance adequately supported by the findings presented in the article?

Yes

Is the rationale for developing the new software tool clearly explained?

Yes

Is the description of the software tool technically sound?

Yes

Are sufficient details of the code, methods and analysis (if applicable) provided to allow replication of the software development and its use by others?

Yes

Is sufficient information provided to allow interpretation of the expected output datasets and any results generated using the tool?

Yes

Reviewer Expertise:

metabolomics, pathway enrichment analyses, multi-omic analyses, bioinformatics

I confirm that I have read this submission and believe that I have an appropriate level of expertise to confirm that it is of an acceptable scientific standard.

# Peer review: Reviewer response for version 2

**DOI:** 10.5256/f1000research.29655.r74950

**Published:** 2021-01-20

**Authors:** Egon L. Willighagen

**Affiliations:** 1 Department of Bioinformatics-BiGCaT, NUTRIM, Maastricht University, Maastricht, The Netherlands

I thank the authors for their detailed response. Overall, I am satisfied with the updates, and where the authors did not agree with me, their reply explains the arguments, which I respect. I do not understand the argument about not changing a dependency as both RCurl and curl are readily available. The open review nature makes the choice clear and that is enough for me.

Are the conclusions about the tool and its performance adequately supported by the findings presented in the article?

Yes

Is the rationale for developing the new software tool clearly explained?

Yes

Is the description of the software tool technically sound?

Yes

Are sufficient details of the code, methods and analysis (if applicable) provided to allow replication of the software development and its use by others?

Yes

Is sufficient information provided to allow interpretation of the expected output datasets and any results generated using the tool?

Yes

Reviewer Expertise:

R packages, identifier mapping, biological pathway resources, metabolomics, cheminformatics

I confirm that I have read this submission and believe that I have an appropriate level of expertise to confirm that it is of an acceptable scientific standard.

# Peer review: Reviewer response for version 1

**DOI:** 10.5256/f1000research.27533.r67235

**Published:** 2020-09-01

**Authors:** Egon L. Willighagen

**Affiliations:** 1 Department of Bioinformatics-BiGCaT, NUTRIM, Maastricht University, Maastricht, The Netherlands

The article describes an R package (gprofiler2) that wraps around the REST interface of a g:Profiler web service. The paper outlines the method used to access the API, and explains that providing an R API makes it easier to integrate with the functionality of other R packages. The description of the software is sufficient and complementary to the source code repository (and backed by a Zenodo archive). The examples given nicely demonstrate the functionality and I tried many of them in rstudio (some comments below). The visualisation, as is most functionality, is inherited from the website, and as such benefits from a larger user base.

 The choice of the combination of having the code base under version control, archiving on Zenodo, and distribution via CRAN shows high-quality standards. A test suite, unfortunately, is missing, and adding it is recommended. Furthermore, it could be interesting to explore curl as a replacement of RCurl, as I had trouble with SSL certificates with the RCurl package on Windows (but not essential).

 I can build the package from source. When applying a regular CRAN check on the package I get a couple of warnings and notes, but none that are affecting this paper. I left two source code suggestions: https://github.com/egonw/r-gprofiler2/pull/1.

 I also like to note that I found out that the interactive plot does not seem to work in RStudio: I only get an empty window.

 Regarding the content of the article, I have the following questions. First, the argument that the computation is done on a central server as an advantage depends on the use case and is not generally true. For example, it requires all information to be shared with the server, which is not possible for everyone that may be interested in the server. As such, one could imagine having a Docker with the webservice and method parameters in the R package to interact with servers with a different domain or IP address. This is, however, just a future feature request.

 Second, regarding the data used in the functionality, I have three questions. How is the ordering of pathways in for KEGG, Reactome, and WikiPathways defined? For Reactome, I can imagine their own hierarchy is used. What about KEGG and WikiPathways? Is the Pathway Ontology used for the latter? A similar question applies when using custom GMT files? The ordering is then defined by the other in the file, I assume? Finally, in the Reproducibility section, but it is not clear to me why version information is not given for the data GO, KEGG, Reactome, etc. I would strongly suggest providing that information too. I would suggest clarifying the first two in the manuscript, but the last one may require additional functionality. Alternatively, the article should be clear that version information is only given for a subset of used resources.

 A third topic I like to ask about is the following: If g:Profile is an ELIXIR RIR, have you consider adding the option to use the Identifiers.org prefixes, e.g. "wikipathways" in addition to "WP"?

 Fourth, the "Use case" uses packages that need to be installed. The keep symmetry with the description of gprofiler2 itself, please consider adding install instructions for these Bioconductor packages.

 Last, I collected a number of small textual suggestions and have annotated these with hypothes.is: https://hyp.is/rE4tRurJEeqMHW_RYqO0xA/f1000research.com/articles/9-709.

 These suggestions include a number of title fixes in the bibliography where article titles are all converted to lower case, which looks weird for names of tools.

Are the conclusions about the tool and its performance adequately supported by the findings presented in the article?

Yes

Is the rationale for developing the new software tool clearly explained?

Yes

Is the description of the software tool technically sound?

Yes

Are sufficient details of the code, methods and analysis (if applicable) provided to allow replication of the software development and its use by others?

Yes

Is sufficient information provided to allow interpretation of the expected output datasets and any results generated using the tool?

Yes

Reviewer Expertise:

R packages, identifier mapping, biological pathway resources, metabolomics, cheminformatics

I confirm that I have read this submission and believe that I have an appropriate level of expertise to confirm that it is of an acceptable scientific standard, however I have significant reservations, as outlined above.

# Response

**Published:** 2020-09-15

**Authors:** Hedi Peterson

**Affiliations:** University of Tartu, Estonia

We thank the reviewer for thorough reading of the manuscript and for the suggestions to improve the gprofiler2 package and the g:Profiler tool itself. Reviewer's comments are in **bold** followed by our responses in regular font.

**The choice of the combination of having the code base under version control, archiving on Zenodo, and distribution via CRAN shows high-quality standards. A test suite, unfortunately, is missing, and adding it is recommended.**

We agree that testing is an important part of software development. However, since the main functionality of the gprofiler2 R package is to wrap POST requests to g:Profiler API, which has an extensive test suite, then we decided to not include additional tests to the package. Nevertheless, we use the R CMD checks for the package every time we submit a new version to CRAN. 

**Furthermore, it could be interesting to explore curl as a replacement of RCurl, as I had trouble with SSL certificates with the RCurl package on Windows (but not essential).**

Thank you for the suggestion. The decision to use the RCurl package was made in 2012 when development started for the first version of the g:Profiler package. Replacing this dependency now can potentially cause issues for current users and external pipelines. Therefore, at the moment, we will keep this dependency as is, but we might consider the change in future updates. We are aware of the issues related to SSL certificates on Windows and due to that we provide access to g:Profiler both with and without SSL. 

**I can build the package from source. When applying a regular CRAN check on the package I get a couple of warnings and notes, but none that are affecting this paper. I left two source code suggestions: https://github.com/egonw/r-gprofiler2/pull/1.**

Thank you for the suggestions, we included the suggested changes. 

**I also like to note that I found out that the interactive plot does not seem to work in RStudio: I only get an empty window.**

It appears that this is a known issue with rendering interactive plotly images in the latest RStudio version (see https://github.com/ropensci/plotly/issues/1712 and https://github.com/rstudio/rstudio/issues/7507, for example). One of the publicly proposed solutions is to reinstall RStudio. We hope this solves the interactivity issue.

**Regarding the content of the article, I have the following questions.**

**First, the argument that the computation is done on a central server as an advantage depends on the use case and is not generally true. For example, it requires all information to be shared with the server, which is not possible for everyone that may be interested in the server. As such, one could imagine having a Docker with the webservice and method parameters in the R package to interact with servers with a different domain or IP address. This is, however, just a future feature request.**

We agree that using a central server is problematic for users who cannot share their data. However, centralising computations enables us to maintain the g:Profiler ecosystem better and guarantees that the results from different access points (web tool, R package, Python package, Galaxy tools, custom API requests) remain consistent, which is beneficial for the users. To alleviate data sharing issue we do not store the input gene lists in g:Profiler unless the user explicitly requests their data to be stored for future access/reference via dedicated short links. This information is provided at the footer of the g:Profiler web page, and will be included within the next version of the R package (0.2.1). In addition, we added the following sentences (underlined) to the manuscript:

“Relying on the central API also simplifies the maintenance of the g:Profiler interfaces and enables the R users to get access to the most up-to-date data without having to download the heavy annotation data files to their own devices. At the same time, g:Profiler respects users’ privacy and does not store input gene lists unless the user explicitly requests their data to be stored for future reference via dedicated short links (see section “Sending analysis from R to g:Profiler web interface”).”

“In this case, the variable multi_gp_link is a character string that corresponds to a stable short-link to these enrichment results: https://biit.cs.ut.ee/gplink/l/0wgtcERnQT. We also note that the input gene lists will be stored in a database to provide short-link access.”

**Second, regarding the data used in the functionality, I have three questions.**

**How is the ordering of pathways in for KEGG, Reactome, and WikiPathways defined? For Reactome, I can imagine their own hierarchy is used. What about KEGG and WikiPathways? Is the Pathway Ontology used for the latter?**

We use the order provided by the corresponding data source for all three of them. For example, pathways from KEGG are provided in the order of their identifiers, but pathways from Reactome are ordered by the name of the pathway term. We do not currently use the Pathway Ontology for WikiPathways. Thus, the only source for which we use hierarchical ontology order in the Manhattan plot is Gene Ontology, but the x-axis positions are fixed for all of the sources. This means that the terms in the Manhattan plot do not change the position along the x-axis when the input query changes. 

**A similar question applies when using custom GMT files? The ordering is then defined by the other in the file, I assume?**

Yes, in case of custom GMT files, the order of the terms in the Manhattan plot is defined by their order in the GMT file. We included the following sentence (underlined) to the manuscript to clarify this:

“The custom data source results can also be plotted using the Manhattan plots (Figure 3). In this case, the term position on the x-axis is defined by the order in the GMT file.”   

**Finally, in the Reproducibility section, but it is not clear to me why version information is not given for the data GO, KEGG, Reactome, etc. I would strongly suggest providing that information too. I would suggest clarifying the first two in the manuscript, but the last one may require additional functionality. Alternatively, the article should be clear that version information is only given for a subset of used resources.**

Historically, g:Profiler versioning schema is based on Ensembl releases because we update our database according to their update schedule (with a time lag). The version fixes the exact extract of g:Profiler annotation database to give a point of reference for g:Profiler enrichment results and is not intended to cover all the numerous data source versions. However, we agree that the source specific version information might be useful for the users and it is available from the g:Profiler’s web page (under the “Data sources” section there is a link “Show data versions”). We included this information to the revised manuscript. Furthermore, based on this suggestion, we also added a new function get_version_info(organism) to the R package that will enable users to obtain the versions of all the data sources used for the set g:Profiler version and organism. The function is already in the source code repository https://gl.cs.ut.ee/biit/r-gprofiler2, but currently works only for the Beta version, i.e. if set_base_url(“ http://biit.cs.ut.ee/gprofiler_beta”). This functionality will be included in the next CRAN release of the gprofiler2 R package (0.2.1).  

The amendments in the section “Reproducibility” to clarify the g:Profiler version info are underlined in the excerpt below. 

“The g:Profiler specific version number notes that the results were obtained using the state of the database that includes data from Ensembl release 99, Ensembl Genomes release 46 and WormBase ParaSite release 14, among other sources, and the g:Profiler codebase with the Git revision number f929183. The version number together with the details of applied parameters (available from multi_gp$meta$query_metadata) is enough to reproduce the enrichment results in g:Profiler. A more detailed information about the data source versions in a given g:Profiler version is available from the g:Profiler web page https://biit.cs.ut.ee/gprofiler under the link “Show data versions” in the “Data sources” section.“ 

**A third topic I like to ask about is the following: If g:Profile is an ELIXIR RIR, have you consider adding the option to use the Identifiers.org prefixes, e.g. "wikipathways" in addition to "WP"?**

We have not considered the option to use the identifiers.org prefixes at the moment. Currently all the terms are linked inside the g:Profiler toolset and no external links are provided. However, we thank the reviewer for this suggestion and we will consider it for further g:Profiler releases.

**Fourth, the "Use case" uses packages that need to be installed. The keep symmetry with the description of gprofiler2 itself, please consider adding install instructions for these Bioconductor packages.**

We included the suggested installation instructions in the revised version of the manuscript. 

**Last, I collected a number of small textual suggestions and have annotated these with hypothes.is: https://hyp.is/rE4tRurJEeqMHW_RYqO0xA/f1000research.com/articles/9-709. These suggestions include a number of title fixes in the bibliography where article titles are all converted to lower case, which looks weird for names of tools.**

Thank you very much for noticing the issues from bibliography parsing. We updated the manuscript according to the suggestions.
